# *N*-(2,3-Dimethyl­phen­yl)succinimide

**DOI:** 10.1107/S160053681001055X

**Published:** 2010-03-24

**Authors:** B. S. Saraswathi, B. Thimme Gowda, Sabine Foro, Hartmut Fuess

**Affiliations:** a Department of Chemistry, Mangalore University, Mangalagangotri 574 199, Mangalore, India; b Institute of Materials Science, Darmstadt University of Technology, Petersenstrasse 23, D-64287 Darmstadt, Germany

## Abstract

In the title compound, C_12_H_13_NO_2_, the dihedral angle between the aromatic benzene ring and the imide segment is 67.7 (1)°. The mol­ecules in the crystal are packed into layered chains along the *c* axis.

## Related literature

For our study of the effect of ring and side-chain substitutions on the structures of biologically significant compounds, see: Gowda *et al.* (2007 ▶); Saraswathi *et al.* (2010**a* ▶,b* ▶).
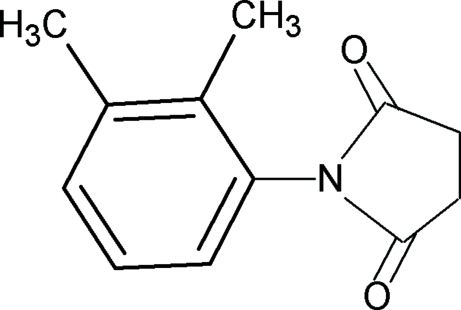


## Experimental

### 

#### Crystal data

C_12_H_13_NO_2_
*M* *_r_* = 203.23
Monoclinic, 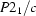

*a* = 6.0600 (5) Å
*b* = 16.429 (2) Å
*c* = 10.593 (1) Å
β = 91.992 (8)°
*V* = 1054.00 (18) Å^3^
*Z* = 4
Cu *K*α radiation
μ = 0.71 mm^−1^
*T* = 299 K
0.40 × 0.20 × 0.15 mm

#### Data collection

Enraf–Nonius CAD-4 diffractometer
2096 measured reflections
1883 independent reflections
1472 reflections with *I* > 2σ(*I*)
*R*_int_ = 0.016
3 standard reflections every 120 min  intensity decay: 1.0%

#### Refinement

*R*[*F* ^2^ > 2σ(*F* ^2^)] = 0.041
*wR*(*F* ^2^) = 0.122
*S* = 1.05
1883 reflections
139 parameters
H-atom parameters constrained
Δρ_max_ = 0.18 e Å^−3^
Δρ_min_ = −0.21 e Å^−3^

### 

Data collection: *CAD-4-PC* (Enraf–Nonius, 1996 ▶); cell refinement: *CAD-4-PC*; data reduction: *REDU4* (Stoe & Cie, 1987 ▶); program(s) used to solve structure: *SHELXS97* (Sheldrick, 2008 ▶); program(s) used to refine structure: *SHELXL97* (Sheldrick, 2008 ▶); molecular graphics: *PLATON* (Spek, 2009 ▶); software used to prepare material for publication: *SHELXL97*.

## Supplementary Material

Crystal structure: contains datablocks I, global. DOI: 10.1107/S160053681001055X/ng2748sup1.cif

Structure factors: contains datablocks I. DOI: 10.1107/S160053681001055X/ng2748Isup2.hkl

Additional supplementary materials:  crystallographic information; 3D view; checkCIF report

## Figures and Tables

**Fig. 1.**
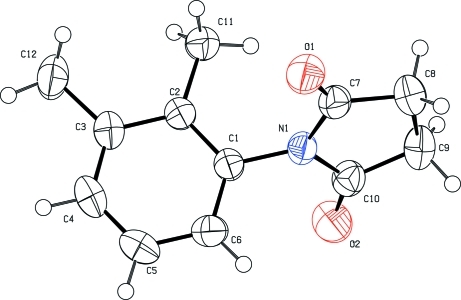
Molecular structure of the title compound, showing the atom labelling scheme. The displacement ellipsoids are drawn at the 50% probability level. The H atoms are represented as small spheres of arbitrary radii.

**Fig. 2.**
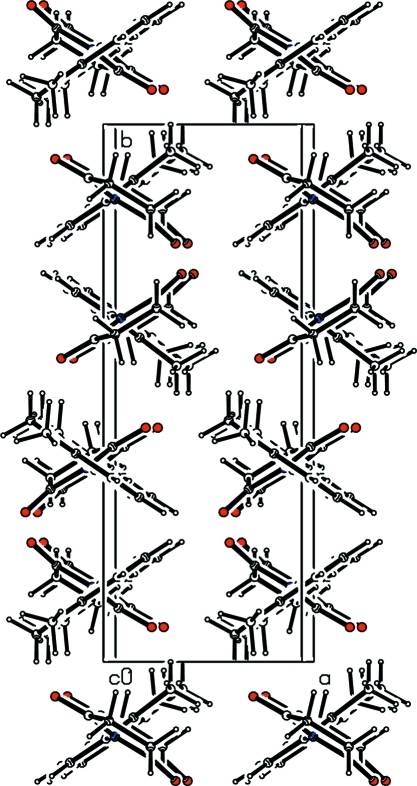
Molecular packing of the title compound.

## supplementary crystallographic information

### Comment

As a part of studying the effect of ring and side chain substitutions on the structures of biologically significant compounds (Gowda *et al.*, 2007; Saraswathi *et al.*, 2010*a,b*), the crystal structure of *N*,*N*-(2,3-dimethylphenyl)succinimide has been determined (Fig. 1). The dihedral angle between the benzene ring and the imide segment in the molecule is 67.7 (1)°, compared to the values of 85.7 (1)° in *N*,*N*-(2,4-dimethylphenyl)succinimide (Saraswathi *et al.*, 2010*b*) and 75.9 (1)° *N*,*N*-(2,6-dimethylphenyl)succinimide (Saraswathi *et al.*, 2010*a*).

The torsion angles of the groups, C2 - C1 - N1 - C7, C6 - C1 - N1 - C7, C2 - C1 - N1 - C10 and C6 - C1 - N1 - C10 in the molecule are 70.2 (2), -109.3 (2), -113.1 (2) and 67.5 (2)°, respectively, while the torsional angles of the groups, O1 - C7 - N1 - C1, C8 - C7 - N1 - C1, O2 - C10 - N1 - C1 and C9 - C10 - N1 - C1 are 2.9 (3), -177.4 (2), 0.2 (3) and -180.0 (2)°, respectively.

The packing of molecules into layered row like chains along *c*-axis is shown in Fig.2.

### Experimental

The solution of succinic anhydride (0.025 mole) in toluene (25 ml) was treated dropwise with the solution of 2,3-dimethylaniline (0.025 mole) also in toluene (25 ml) with constant stirring. The resulting mixture was stirred for one h and set aside for an additional hour at room temperature for the completion of reaction. The mixture was then treated with dilute hydrochloric acid to remove the unreacted 2,3-dimethylaniline. The resultant solid *N*-(2,3-dimethylphenyl)succinamic acid was filtered under suction and washed thoroughly with water to remove the unreacted succinic anhydride and succinic acid. It was recrystallized to constant melting point from ethanol.

*N*-(2,3-Dimethylphenyl)succinamic acid was heated for 2 h and then allowed to cool slowly to room temperature to get the compound, *N*-(2,3-dimethylphenyl)succinimide. The purity of the compound was checked and characterized by its infrared spectra.

Prism like colourless single crystals of the compound used in X-ray diffraction studies were grown in ethanolic solution by a slow evaporation at room temperature.

### Refinement

The H atoms were positioned with idealized geometry using a riding model with C—H = 0.93–0.97 Å. Isotropic displacement parameters for the H atoms were set equal to 1.2 U_eq_ (parent atom).

In the absence of significant anomalous dispersion effects, Friedel pairs were merged and the Δf"term set to zero.

### Crystal data

**Table:**

C_12_H_13_NO_2_	*F*(000) = 432
*M**_r_* = 203.23	*D*_x_ = 1.281 Mg m^−^^3^
Monoclinic, *P*2_1_/*c*	Cu *K*α radiation, λ = 1.54180 Å
Hall symbol: -P 2ybc	Cell parameters from 25 reflections
*a* = 6.0600 (5) Å	θ = 5.4–18.0°
*b* = 16.429 (2) Å	µ = 0.71 mm^−^^1^
*c* = 10.593 (1) Å	*T* = 299 K
β = 91.992 (8)°	Prism, colourless
*V* = 1054.00 (18) Å^3^	0.40 × 0.20 × 0.15 mm
*Z* = 4	

### Data collection

**Table:**

Enraf–Nonius CAD-4 diffractometer	*R*_int_ = 0.016
Radiation source: fine-focus sealed tube	θ_max_ = 66.9°, θ_min_ = 5.0°
graphite	*h* = −7→1
ω/2θ scans	*k* = −19→0
2096 measured reflections	*l* = −12→12
1883 independent reflections	3 standard reflections every 120 min
1472 reflections with *I* > 2σ(*I*)	intensity decay: 1.0%

### Refinement

**Table:**

Refinement on *F*^2^	Secondary atom site location: difference Fourier map
Least-squares matrix: full	Hydrogen site location: inferred from neighbouring sites
*R*[*F*^2^ > 2σ(*F*^2^)] = 0.041	H-atom parameters constrained
*wR*(*F*^2^) = 0.122	*w* = 1/[σ^2^(*F*_o_^2^) + (0.0595*P*)^2^ + 0.2594*P*] where *P* = (*F*_o_^2^ + 2*F*_c_^2^)/3
*S* = 1.05	(Δ/σ)_max_ < 0.001
1883 reflections	Δρ_max_ = 0.18 e Å^−^^3^
139 parameters	Δρ_min_ = −0.20 e Å^−^^3^
0 restraints	Extinction correction: *SHELXL97* (Sheldrick, 2008), Fc^*^=kFc[1+0.001xFc^2^λ^3^/sin(2θ)]^-1/4^
Primary atom site location: structure-invariant direct methods	Extinction coefficient: 0.0081 (9)

### Special details

**Table:**

Geometry. All esds (except the esd in the dihedral angle between two l.s. planes) are estimated using the full covariance matrix. The cell esds are taken into account individually in the estimation of esds in distances, angles and torsion angles; correlations between esds in cell parameters are only used when they are defined by crystal symmetry. An approximate (isotropic) treatment of cell esds is used for estimating esds involving l.s. planes.
Refinement. Refinement of *F*^2^ against ALL reflections. The weighted *R*-factor wR and goodness of fit *S* are based on *F*^2^, conventional *R*-factors *R* are based on *F*, with *F* set to zero for negative *F*^2^. The threshold expression of *F*^2^ > σ(*F*^2^) is used only for calculating *R*-factors(gt) etc. and is not relevant to the choice of reflections for refinement. *R*-factors based on *F*^2^ are statistically about twice as large as those based on *F*, and *R*- factors based on ALL data will be even larger.

### Fractional atomic coordinates and isotropic or equivalent isotropic displacement parameters (Å^2^)

**Table:**

	*x*	*y*	*z*	*U*_iso_*/*U*_eq_	
C1	0.0295 (3)	0.64329 (10)	0.78424 (14)	0.0372 (4)	
C2	0.1636 (3)	0.60330 (10)	0.70029 (15)	0.0376 (4)	
C3	0.1134 (3)	0.61237 (11)	0.57082 (15)	0.0452 (4)	
C4	−0.0697 (3)	0.65748 (12)	0.53242 (17)	0.0546 (5)	
H4	−0.1026	0.6632	0.4465	0.066*	
C5	−0.2041 (3)	0.69410 (12)	0.61716 (19)	0.0557 (5)	
H5	−0.3281	0.7230	0.5888	0.067*	
C6	−0.1541 (3)	0.68768 (11)	0.74460 (18)	0.0467 (4)	
H6	−0.2424	0.7128	0.8031	0.056*	
C7	0.2655 (3)	0.67879 (11)	0.97290 (16)	0.0449 (4)	
C8	0.2745 (3)	0.65793 (14)	1.11109 (17)	0.0579 (5)	
H8A	0.2780	0.7069	1.1622	0.069*	
H8B	0.4045	0.6256	1.1324	0.069*	
C9	0.0665 (4)	0.60978 (13)	1.13227 (17)	0.0567 (5)	
H9A	0.1021	0.5571	1.1689	0.068*	
H9B	−0.0287	0.6388	1.1887	0.068*	
C10	−0.0448 (3)	0.59991 (11)	1.00467 (17)	0.0461 (4)	
C11	0.3529 (3)	0.55084 (11)	0.74623 (17)	0.0478 (5)	
H11A	0.3393	0.5398	0.8346	0.057*	
H11B	0.4896	0.5787	0.7332	0.057*	
H11C	0.3509	0.5005	0.7002	0.057*	
C12	0.2559 (4)	0.57447 (15)	0.47345 (18)	0.0657 (6)	
H12A	0.2417	0.5163	0.4767	0.079*	
H12B	0.4072	0.5894	0.4905	0.079*	
H12C	0.2098	0.5935	0.3910	0.079*	
N1	0.0830 (2)	0.63939 (9)	0.91715 (12)	0.0393 (4)	
O1	0.3887 (2)	0.72166 (9)	0.91606 (13)	0.0598 (4)	
O2	−0.2143 (2)	0.56450 (9)	0.97796 (13)	0.0629 (4)	

### Atomic displacement parameters (Å^2^)

**Table:**

	*U*^11^	*U*^22^	*U*^33^	*U*^12^	*U*^13^	*U*^23^
C1	0.0369 (8)	0.0402 (9)	0.0343 (8)	−0.0050 (7)	0.0002 (6)	0.0017 (7)
C2	0.0378 (8)	0.0377 (8)	0.0372 (8)	−0.0035 (7)	0.0002 (7)	0.0019 (7)
C3	0.0537 (10)	0.0461 (10)	0.0357 (9)	−0.0048 (8)	0.0001 (7)	0.0012 (7)
C4	0.0657 (12)	0.0582 (12)	0.0387 (10)	−0.0021 (10)	−0.0131 (9)	0.0073 (9)
C5	0.0473 (10)	0.0570 (12)	0.0618 (12)	0.0069 (9)	−0.0129 (9)	0.0082 (9)
C6	0.0387 (9)	0.0495 (10)	0.0519 (10)	0.0029 (8)	0.0027 (8)	0.0018 (8)
C7	0.0419 (9)	0.0512 (11)	0.0415 (9)	−0.0016 (8)	0.0015 (7)	−0.0083 (8)
C8	0.0617 (12)	0.0701 (13)	0.0411 (10)	−0.0007 (10)	−0.0073 (9)	−0.0074 (9)
C9	0.0758 (14)	0.0576 (12)	0.0369 (10)	−0.0013 (10)	0.0049 (9)	0.0013 (9)
C10	0.0510 (11)	0.0458 (10)	0.0417 (9)	0.0011 (8)	0.0053 (8)	0.0052 (8)
C11	0.0466 (10)	0.0521 (11)	0.0449 (10)	0.0058 (8)	0.0032 (8)	0.0025 (8)
C12	0.0805 (15)	0.0784 (15)	0.0385 (10)	0.0053 (12)	0.0067 (10)	−0.0076 (10)
N1	0.0396 (7)	0.0456 (8)	0.0328 (7)	−0.0029 (6)	0.0031 (6)	0.0000 (6)
O1	0.0493 (8)	0.0726 (10)	0.0576 (8)	−0.0187 (7)	0.0058 (6)	−0.0073 (7)
O2	0.0546 (8)	0.0747 (10)	0.0592 (8)	−0.0185 (7)	0.0015 (7)	0.0170 (7)

### Geometric parameters (Å, °)

**Table:**

C1—C6	1.383 (2)	C8—C9	1.512 (3)
C1—C2	1.390 (2)	C8—H8A	0.9700
C1—N1	1.435 (2)	C8—H8B	0.9700
C2—C3	1.402 (2)	C9—C10	1.498 (3)
C2—C11	1.502 (2)	C9—H9A	0.9700
C3—C4	1.384 (3)	C9—H9B	0.9700
C3—C12	1.503 (3)	C10—O2	1.206 (2)
C4—C5	1.372 (3)	C10—N1	1.389 (2)
C4—H4	0.9300	C11—H11A	0.9600
C5—C6	1.377 (3)	C11—H11B	0.9600
C5—H5	0.9300	C11—H11C	0.9600
C6—H6	0.9300	C12—H12A	0.9600
C7—O1	1.203 (2)	C12—H12B	0.9600
C7—N1	1.395 (2)	C12—H12C	0.9600
C7—C8	1.503 (3)		
			
C6—C1—C2	122.47 (15)	H8A—C8—H8B	108.8
C6—C1—N1	118.25 (15)	C10—C9—C8	105.92 (15)
C2—C1—N1	119.28 (14)	C10—C9—H9A	110.6
C1—C2—C3	117.62 (15)	C8—C9—H9A	110.6
C1—C2—C11	121.38 (15)	C10—C9—H9B	110.6
C3—C2—C11	120.98 (15)	C8—C9—H9B	110.6
C4—C3—C2	119.26 (17)	H9A—C9—H9B	108.7
C4—C3—C12	119.62 (17)	O2—C10—N1	123.97 (17)
C2—C3—C12	121.12 (17)	O2—C10—C9	128.09 (17)
C5—C4—C3	122.07 (17)	N1—C10—C9	107.93 (15)
C5—C4—H4	119.0	C2—C11—H11A	109.5
C3—C4—H4	119.0	C2—C11—H11B	109.5
C4—C5—C6	119.49 (17)	H11A—C11—H11B	109.5
C4—C5—H5	120.3	C2—C11—H11C	109.5
C6—C5—H5	120.3	H11A—C11—H11C	109.5
C5—C6—C1	119.02 (17)	H11B—C11—H11C	109.5
C5—C6—H6	120.5	C3—C12—H12A	109.5
C1—C6—H6	120.5	C3—C12—H12B	109.5
O1—C7—N1	123.82 (16)	H12A—C12—H12B	109.5
O1—C7—C8	128.24 (17)	C3—C12—H12C	109.5
N1—C7—C8	107.95 (15)	H12A—C12—H12C	109.5
C7—C8—C9	105.16 (15)	H12B—C12—H12C	109.5
C7—C8—H8A	110.7	C10—N1—C7	112.72 (14)
C9—C8—H8A	110.7	C10—N1—C1	124.29 (14)
C7—C8—H8B	110.7	C7—N1—C1	122.92 (14)
C9—C8—H8B	110.7		
			
C6—C1—C2—C3	3.0 (2)	C7—C8—C9—C10	3.9 (2)
N1—C1—C2—C3	−176.40 (15)	C8—C9—C10—O2	178.99 (19)
C6—C1—C2—C11	−175.79 (16)	C8—C9—C10—N1	−0.8 (2)
N1—C1—C2—C11	4.8 (2)	O2—C10—N1—C7	177.23 (18)
C1—C2—C3—C4	−2.4 (2)	C9—C10—N1—C7	−2.9 (2)
C11—C2—C3—C4	176.39 (17)	O2—C10—N1—C1	0.2 (3)
C1—C2—C3—C12	177.13 (18)	C9—C10—N1—C1	−179.96 (16)
C11—C2—C3—C12	−4.1 (3)	O1—C7—N1—C10	−174.22 (17)
C2—C3—C4—C5	0.2 (3)	C8—C7—N1—C10	5.5 (2)
C12—C3—C4—C5	−179.3 (2)	O1—C7—N1—C1	2.9 (3)
C3—C4—C5—C6	1.5 (3)	C8—C7—N1—C1	−177.41 (15)
C4—C5—C6—C1	−0.9 (3)	C6—C1—N1—C10	67.5 (2)
C2—C1—C6—C5	−1.4 (3)	C2—C1—N1—C10	−113.08 (19)
N1—C1—C6—C5	178.08 (16)	C6—C1—N1—C7	−109.27 (19)
O1—C7—C8—C9	174.0 (2)	C2—C1—N1—C7	70.2 (2)
N1—C7—C8—C9	−5.7 (2)		
